# Effects of Smoking on the Sleep-Wakefulness Cycle: Actigraphic Research Among University Students

**DOI:** 10.7759/cureus.115079

**Published:** 2026-08-24

**Authors:** Ali F Banafa, Arusha Ayub, Abdullah F Banafa, Marwan Jaber, Saraha M Ansari, Ahmed Khalid, Parvin Mozafari, Tazeen A Kadri, Yukta P Anchan, Waleed Qasem, Mariam Gogichadze

**Affiliations:** 1 School of Health Sciences, University of Georgia, Tbilisi, GEO

**Keywords:** actigraphy, circadian rhythm, nicotine dependence, sleep-wake cycle, smoking, university students

## Abstract

Introduction: Sleep is one of the most important biological processes of human beings, and its functions are essential to cognitive and emotional as well as mental and physical health. Nicotine, a chemical stimulant in cigarettes and e-cigarettes, acts both as a stimulant and a relaxant on the human central nervous system. Cigarette and e-cigarette smoking may not only delay sleep onset and reduce total sleep time but also alter the normal sleep stage architecture. In addition to lower academic performance, inadequate sleep quality and disrupted circadian rhythms are also known to cause various other poor health outcomes in both physical and mental health, including increases in chronic disease, especially in university students. This study aimed to assess the effect of cigarette and e-cigarette smoking on the sleep-wakefulness cycle in students of the University of Georgia, Tbilisi, objectively, using wearable actigraphy. Questionnaires were collected to assess the same effects subjectively.

Methods: This study design was a cross-sectional observational design of 18-25-year-old university students categorized into current cigarette/e-cigarette users (smokers) and current non-smokers who reported at least one year without smoking/vaping. The data for the sleep-wakefulness parameters were collected using wrist-worn wearable devices manufactured by Huawei Technologies Co., Ltd. and Zepp Health Corporation and measured for seven continuous days of the total sleep duration, rapid eye movement sleep duration (REM-SD), deep sleep duration, and number of nocturnal awakenings. A questionnaire was distributed via Google Forms to university students to assess their sleep patterns and associated behaviors. Sleep-wakefulness data were then statistically analyzed using IBM SPSS Statistics version 23.0. An independent-samples t-test was used to compare parameters between smokers and non-smokers; p <0.05 was considered statistically significant.

Results: A total of 47 smokers and 50 non-smokers had successfully collected their sleep-wakefulness data using an actigraphy device. A total of 107 responses from the surveys were collected, with 96 smokers and 11 non-smokers. The sleep parameters results showed that total sleep time was significantly shorter in smokers compared to non-smokers (381.60±163.22 minutes vs. 410.37±133.71 minutes, p = 0.012), whereas REM-SD was also significantly reduced in smokers compared to non-smokers (55.09± 57.55 minutes vs. 63.89±39.94 minutes, p = 0.020). There was no significant difference in deep sleep duration between smokers and non-smokers (81.10 vs. 60.79 minutes vs. 88.78 vs. 45.89 minutes, p = 0.061). The survey data showed that many smokers had smoked a cigarette close to bedtime, slept lighter, took a longer time to sleep, woke up frequently during the night, and felt fatigued during the morning.

Conclusion: Our study results found that cigarette and e-cigarette smoking may cause poorer sleep quality by altering two of the main sleep parameters. Nicotine usage leads to significant reductions in sleep quality parameters, including total sleep time and REM-SD, and thus it may also adversely affect other physiological benefits, including improved mental health and physical health. Therefore, programs focused on health education may target smokers who are involved in these poor sleep characteristics as one of the interventions to help them with cessation and to improve their overall well-being.

## Introduction

The most important physiological process for physical and mental well-being is sleep [1]. Negative outcomes that result from the short length of time slept, poor sleep quality, and sleep disturbances include impaired performance and the failure to remember information, reduced academic achievement, and an overall poor life quality [2]. High school and college students are at increased risk of the problems resulting from poor sleep due to the intense work of the education system and a lifestyle that often leads to insufficient amounts of sleep [1]. The utilization of tobacco and nicotine products by young adults and its link to poor sleep health is a widely acknowledged and known risk factor [3]. The exact pathway and extent to which it relates needs to be studied to develop successful public health initiatives [4].

The mechanism that connects nicotine use to sleep disturbances is primarily attributed to the pharmacological effects of nicotine as a strong stimulant of the central nervous system [5]. Nicotine affects the release of crucial neurotransmitters, such as dopamine, norepinephrine, and acetylcholine, which play a critical role in the sleep-wake cycle. As a result of these stimulating effects, there is an increase in physiological arousal, which increases sleep onset latency, which is the amount of time it takes for a person to fall asleep after attempting to sleep, and sleep continuity, which indicates the ability to maintain uninterrupted sleep throughout the night [6]. Due to the fast metabolism of nicotine, there are instances of nicotine withdrawal at night [4]. Nighttime awakenings and increased wakefulness after sleep onset, the total amount of time a person spends awake after initially falling asleep and before the final awakening, can be attributed to the symptoms of restlessness and cravings experienced during nicotine withdrawal. These disturbances have been confirmed through polysomnography (PSG), a form of objective assessment of sleep architecture, among smokers who exhibit shorter total sleep times, lower sleep efficiency, and altered sleep architecture compared to non-smokers, such as reductions in slow-wave sleep [5]. Even passive exposure to tobacco smoke (secondhand smoke) may be related to a greater incidence of sleep disturbance symptoms in adolescents [7].

There are currently a large number of cross-sectional studies relying on subjective assessments of sleep quality. In multiple studies using such instruments as the Insomnia Severity Index (ISI) and the Pittsburgh Sleep Quality Index (PSQI), it has been found that smokers report worse sleep quality than non-smokers [3]. In large population-based health surveys in numerous countries, it was reported that those who smoked had low sleep quality [8]. Similarly, in a college-based study, smokers were found to be more likely to experience a longer delay in sleep onset and more sleep disturbances [9]. Since sleep disturbances are associated with the degree of nicotine dependency, there may be a link between physical nicotine dependency and sleep disturbances [10].

Recently, the widespread use of electronic nicotine delivery systems (ENDS) has become of increasing interest. Studies on adolescents have reported that users of electronic cigarettes (e-cigarettes) report sleep disturbances more often than non-users [11]. It was also reported that among product users, those who smoke combustible cigarettes and ENDS simultaneously (dual users) had the highest risk of failing to meet the less than seven hours per night standard sleep duration [12]. Therefore, nicotine consumption, regardless of product type, is positively correlated with poor sleep quality [11]. This is consistent with earlier studies that examine the effect of different drugs on the sleep-wake cycle, which refers to the regular pattern of alternating wakefulness and sleep over a 24-hour period [13].

Sleep is a dynamic physiological process composed of non-rapid eye movement (NREM) sleep and rapid eye movement (REM) sleep. NREM sleep includes stages N1, N2, and N3 and constitutes the majority of total sleep. It is characterized by progressively slower brain activity, reduced physiological activity, and decreasing responsiveness to external stimuli [5]. Stage N1 is the transition between wakefulness and sleep and usually lasts one to seven minutes. Stage N2 is the most common stage of sleep, accounting for approximately 45%-55% of total sleep. Stage N3 is the deepest stage of NREM sleep and is often called slow-wave sleep because of the predominance of delta waves (0.5-2 Hz). REM sleep is characterized by rapid eye movements, vivid dreaming, increased brain activity, and skeletal muscle atonia. Despite the body's temporary paralysis, the brain remains highly active [7].

Sleep is also characterized by distinct patterns of electrical brain activity known as brain waves, which are measured using electroencephalography (EEG). The type and frequency of these brain waves change as an individual progresses through the different stages of sleep and reflect the depth and quality of sleep. Alpha waves (8-13 Hz) are present during relaxed wakefulness, particularly when a person is awake with their eyes closed but not yet asleep. As an individual begins to fall asleep, alpha activity gradually decreases and is replaced by slower brain wave patterns [2]. During Stage N1 sleep, theta waves (4-7 Hz) become the dominant brain wave. Theta activity represents the transition from wakefulness to sleep and is associated with light sleep, during which a person can still be awakened easily. In Stage N2 sleep, two characteristic EEG features appear: sleep spindles and K-complexes. Sleep spindles are brief bursts of rhythmic brain activity ranging from approximately 11-16 Hz and are believed to play an important role in memory consolidation, learning, and protecting sleep from external disturbances. K-complexes are large, high-amplitude waveforms that occur either spontaneously or in response to external stimuli and help maintain sleep by suppressing unnecessary arousals. During Stage N3, also known as slow-wave sleep, the EEG is dominated by delta waves (0.5-2 Hz) [3]. Delta waves are high-amplitude, low-frequency brain waves that indicate the deepest stage of sleep. This stage is essential for physical restoration, tissue repair, growth hormone secretion, immune function, and recovery from daily physical and mental activity. In REM sleep, brain activity changes markedly. The EEG displays low-amplitude, mixed-frequency waves, predominantly beta waves (13-30 Hz) along with characteristic sawtooth waves. Although the brain activity during REM sleep closely resembles that of wakefulness, the body experiences temporary muscle paralysis (muscle atonia), preventing the individual from acting out dreams. REM sleep is strongly associated with vivid dreaming, emotional regulation, learning, and memory consolidation [6].

Despite the abundant subjective evidence, there remains a notable deficiency in the objective, ecologically valid measurement of student sleep quality and consistency. Objective metrics of the time spent in the most deeply restorative sleep, the duration of slow wave sleep, and the frequency of micro-arousals, brief, transient awakenings or shifts toward wakefulness that occur during sleep, typically lasting three to 15 seconds, cannot be adequately measured using subjective, memory-based questionnaires because of recall bias [3]. Although PSG can provide a comprehensive overview of the structure of sleep, its inability to accurately measure typical sleep-wake cycles over several days in a natural environment is restrictive [5]. To fill this methodological gap, in the current study we utilized wrist-worn actigraph devices to obtain objective sleep data. Students completed seven days of consecutive actigraph monitoring to allow for an assessment of sleep regularity and disruptions. The analysis of the data specifically examined objectively measured levels of alertness, time spent in deep sleep, and sleep patterns. A questionnaire was distributed via Google Forms (Google LLC, Mountain View, CA, USA) to university students to assess their sleep patterns and associated behaviors (Appendix A). We aimed to establish an accurate and robust picture of the relationship between nicotine use and the sleep-wake cycle using objective measurements such as these and relating them to the actual nicotine use status of college students to inform the design of targetable smoking cessation interventions and sleep health education programs [14].

## Materials and methods

Study design

This study employed a cross-sectional observational comparative design to investigate the effects of smoking on sleep-wakefulness patterns among university students. The research was structured using the Population, Exposure, Comparator, and Outcome (PECO) framework. The population comprised university students aged 18-25 years. The intervention was active smoking behavior, encompassing cigarette and e-cigarette (vaping) use, including frequency, duration, product type, and timing of use relative to bedtime. The comparator consisted of non-smokers or students who had abstained from all smoking or vaping for a minimum of one year. The outcomes of interest were actigraphy-derived sleep-wake cycle parameters, specifically total sleep duration, deep sleep duration, REM sleep duration (REM-SD), frequency of nocturnal awakenings, and overall sleep continuity. Data collection was conducted from May 4, 2025, to December 7, 2025. The actigraphy cohort underwent wrist-based actigraphy monitoring for seven consecutive days per participant, while the independent questionnaire cohort completed the questionnaire during the same overall data-collection period.

Eligibility criteria

Eligibility criteria were defined separately for the actigraphy and independent questionnaire cohorts. The general eligibility criteria were the same for both cohorts, with the exception that the actigraphy cohort had an additional requirement to be able and willing to wear a wrist-based actigraphy device continuously for seven consecutive days.

For both cohorts, inclusion criteria were full-time university student status, age between 18 and 25 years, and either current regular use of cigarettes or e-cigarettes for the smoker group or no current or prior smoking history for at least one year for the non-smoker group. For participants in the actigraphy cohort, an additional inclusion criterion was the ability and willingness to wear a wrist-based actigraphy device continuously for seven consecutive days.

Exclusion criteria for both cohorts included: part-time or non-university student status; age younger than 18 years or older than 25 years; smoking status that did not meet the predefined criteria for either the smoker or non-smoker group; current use of illicit drugs (e.g., cannabis, cocaine, methamphetamine, or opioids); alcohol consumption or dependence; current use of medications known to affect sleep or circadian rhythms (e.g., sedatives, stimulants, antidepressants, or corticosteroids); a self-reported diagnosis of a sleep disorder (e.g., insomnia, obstructive sleep apnea, narcolepsy, or restless legs syndrome); current shift work or rotating night-shift employment; pregnancy or breastfeeding; participation during periods of unusually high academic or psychological stress or following a major stressful life event that could likely affect normal sleep patterns; and incomplete or unusable study data.

For the actigraphy cohort specifically, inability or unwillingness to wear the actigraphy device continuously for seven consecutive days was also an exclusion criterion. In addition, participants who did not provide sufficient valid actigraphy recordings were excluded from the final actigraphy analysis according to the predefined data-quality criteria. The questionnaire-only cohort was not subject to actigraphy-specific eligibility or data-quality requirements.

Participants and sampling

The actigraphy cohort consisted of full-time university students aged 18-25 years enrolled at the University of Georgia, Tbilisi, Georgia. Convenience sampling was employed, with recruitment conducted through institutional email communications, campus poster advertisements, and classroom announcements. A target sample of approximately 50 smokers and 51 non-smokers was established based on feasibility considerations. Following data screening, three participants in the smoker group and one participant in the non-smoker group were excluded due to insufficient actigraphy recordings, resulting in a final analytic sample of 47 smokers and 50 non-smokers (total N = 97). Potential confounding variables considered included age, sex, caffeine consumption, alcohol use, physical activity level, academic stress, and pre-sleep screen time.

Independent questionnaire cohort

The independent questionnaire cohort consisted of 107 university students recruited separately from the actigraphy cohort across 20 universities. Convenience sampling was employed for recruitment. The questionnaire was administered electronically using Google Forms and distributed to university students through online channels (Appendix A). Participants completed the questionnaire voluntarily and were not part of the actigraphy cohort or subjected to actigraphy monitoring. The questionnaire data were analyzed independently and were used as complementary self-reported evidence alongside the actigraphy findings.

Data collection procedures

Data were collected through two independent components: an actigraphy-based assessment and a separate questionnaire-based survey. For the actigraphy cohort, participants wore wrist-worn actigraphy devices continuously for seven consecutive days and completed a brief questionnaire to collect demographic, lifestyle, and clinical information relevant to sleep assessment and to identify factors that could affect eligibility or sleep-related outcomes. The questionnaire used for the independent survey cohort was not subjected to a formal pilot study or structured expert evaluation prior to data collection; therefore, formal questionnaire validity and reliability measures were not assessed.

Separately, an independent sample of 107 university students completed a structured self-report questionnaire. These participants were not part of the actigraphy cohort and did not undergo actigraphy monitoring. The questionnaire collected demographic information, including age and sex; smoking-related characteristics, including smoking status, product type, quantity, frequency, and duration of use; self-reported sleep characteristics, including total sleep duration, deep sleep, REM sleep, and nocturnal awakenings; and lifestyle and health-related factors, including caffeine and alcohol intake, physical activity level, use of sleep-inducing medications, relevant medical conditions, and perceived stress. The questionnaire data were analyzed independently and were used as complementary self-reported evidence alongside the actigraphy findings.

Actigraphy device and wear protocol

Actigraphy data were recorded using commercially available wrist-worn smartwatch devices, specifically manufactured by Huawei Technologies Co., Ltd. (Shenzhen, China) and Zepp Health Corporation (Hefei, China). Participants were instructed to wear the device continuously for seven consecutive days and nights on the non-dominant wrist, removing it only for bathing, swimming, or charging. During charging periods, participants were advised to disable the Bluetooth connection. All participants were instructed to maintain their habitual daily routines throughout the monitoring period and were specifically asked not to alter their caffeine intake, smoking behavior, or physical activity levels during the study. Daily compliance was assessed through device activity logs and participant self-report.

Actigraphy data acquisition and parameters

The wearable devices continuously recorded movement-based activity and sleep-related parameters using proprietary manufacturer algorithms. Data were synchronized daily with the corresponding mobile application, the Huawei Health application for Huawei device users and the Zepp application for Zepp device users, and exported in raw format for subsequent analysis. Each participant contributed seven nights of actigraphy data, ensuring adequate representation of habitual sleep patterns. Four primary sleep parameters were derived for each recorded night and subsequently averaged across the seven-day monitoring period: total sleep duration, defined as the total time spent asleep per night expressed in hours and minutes; REM-SD, defined as the total duration of rapid eye movement sleep in minutes; deep sleep duration, defined as the total duration of slow-wave sleep in minutes; and nocturnal awakening frequency, defined as the number of awakenings recorded per night. Weekly mean values for each parameter were used in all statistical analyses.

Data processing and quality control

Raw actigraphy data were screened for completeness and physiological plausibility prior to analysis. Nights with prolonged non-wear periods or implausible values were excluded from analysis. Weekly mean values were calculated only for participants with a minimum of five valid nights of recording. Data consistency and integrity were verified prior to all statistical testing.

Statistical analysis

All statistical analyses were performed using IBM SPSS Statistics for Windows, Version 23.0 (IBM Corp., Armonk, NY, USA). Continuous variables were expressed as mean ± standard deviation (SD). Between-group comparisons of actigraphy-derived sleep parameters, total sleep duration, REM-SD, deep sleep duration, and nocturnal awakening frequency between smokers and non-smokers were conducted using the independent-samples t-test. All tests were two-tailed, and a p-value of less than 0.05 was considered statistically significant.

Ethical considerations

This study was conducted under the ethical principles outlined in the Declaration of Helsinki. Ethical approval was obtained from the Institutional Review Board of the University of Georgia, Tbilisi, Georgia (approval reference number: UGREC-07-25). All participants provided written informed consent prior to enrollment. Participants were assured of data confidentiality and were informed of their right to withdraw from the study at any time without consequence or penalty.

## Results

Study sample and data inclusion

A total of 101 participants initially underwent wearable actigraphy monitoring, including 50 smokers and 51 non-smokers. During data screening, three participants from the smoker group were excluded because of insufficient device compliance, prolonged non-wear periods, or incomplete nightly recordings. The final actigraphy analysis therefore included 47 smokers and 50 non-smokers.

All included participants had valid sleep recordings across the monitoring period, allowing reliable calculation of weekly mean values for all evaluated sleep parameters.

Actigraphy-measured total sleep duration

**Figure 1:**
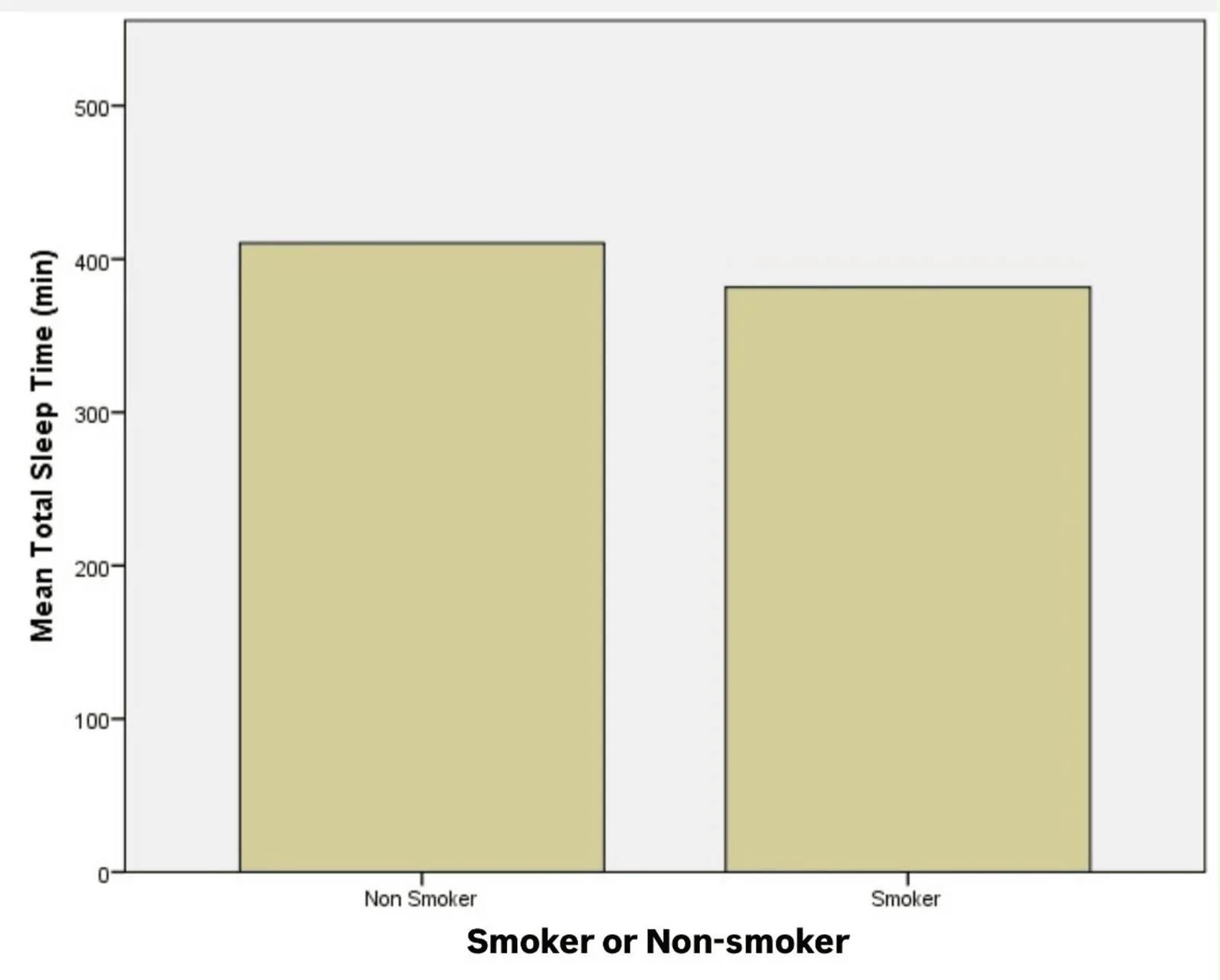
Comparison of mean total sleep duration between smokers and non-smokers

Non-smokers demonstrated a mean total sleep duration of 410.37 ± 133.71 minutes, whereas smokers demonstrated a mean total sleep duration of 381.60 ± 163.22 minutes (Figure 1). Independent-samples t-test analysis demonstrated a statistically significant difference between the two groups (t (95) =2.533, p=0.012), with a mean difference of 28.76 minutes. The 95% CI for the mean difference ranged from 6.47 to 51.06 minutes (Table 1).

**Table 1:** Actigraphy-measured sleep parameters in smokers and non-smokers (with 95% CIs) REM: rapid eye movement

Parameter	Non-smokers (n = 50) Mean ± SD	Smokers (n = 47) Mean ± SD	t(95)	p-value	95% CI for Mean Difference
Total sleep (minutes)	410.37 ± 133.71	381.60 ± 163.22	2.533	0.012	6.47 to 51.06
REM sleep (minutes)	63.89 ± 39.94	55.09 ± 57.55	2.341	0.020	1.42 to 16.18
Deep sleep (minutes)	88.78 ± 45.89	81.10 ± 60.79	1.875	0.061	–0.36 to 15.71
Nocturnal awakenings	Not available	Not available	—	—	—

These findings indicate that smoking status was associated with reduced objectively measured nightly total sleep duration.

Actigraphy-measured REM sleep

**Figure 2:**
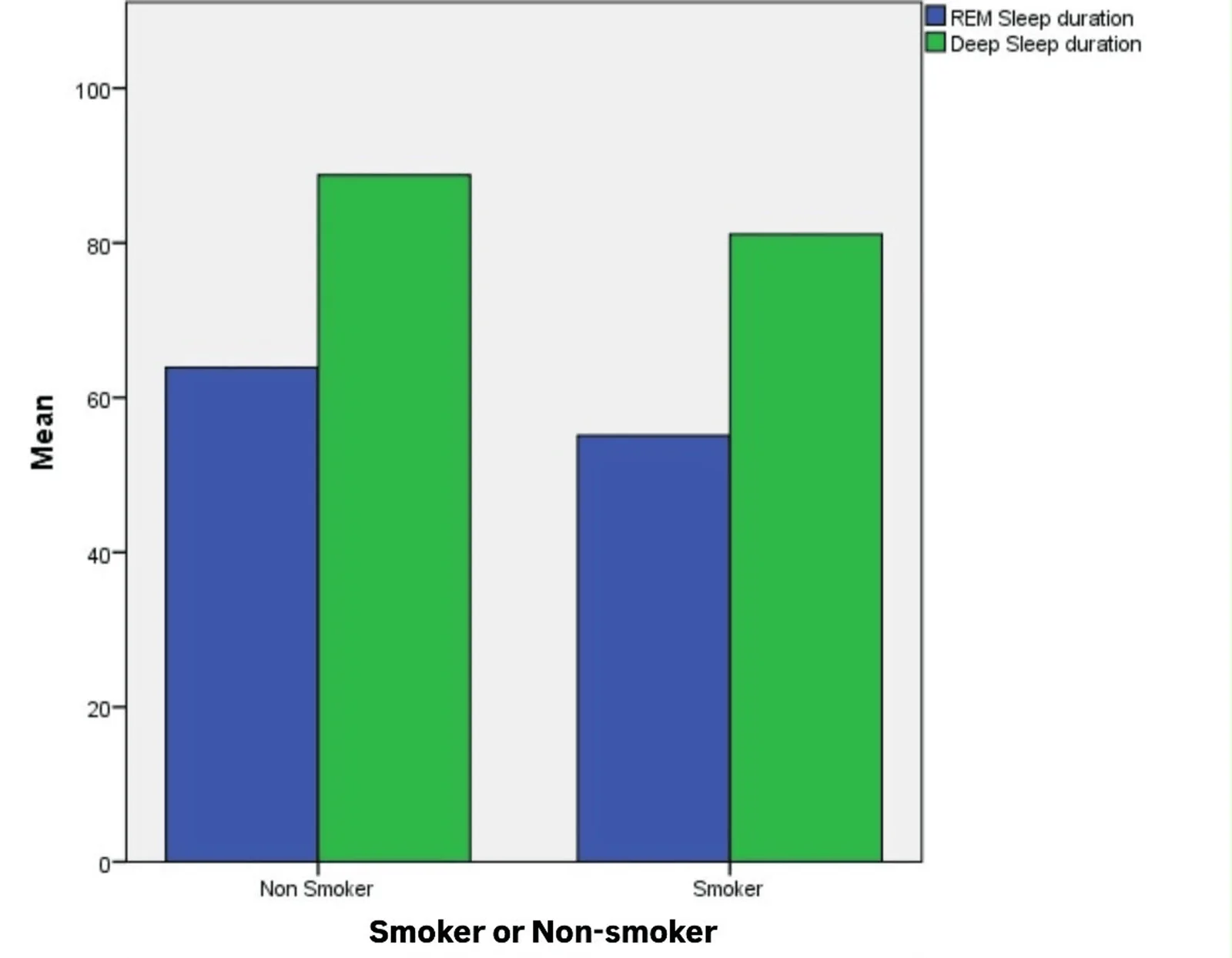
Comparison of mean REM sleep and deep sleep duration between smokers and non-smokers REM: rapid eye movement

The mean REM sleep duration was 63.89 ± 39.94 minutes among non-smokers and 55.09 ± 57.55 minutes among smokers (Figure 2). This difference was statistically significant (t(95)=2.341, p=0.020), with a mean difference of 8.80 minutes. The 95% CI for the mean difference ranged from 1.42 to 16.18 minutes (Table 1).

These findings suggest that smoking status was associated with reduced REM-SD as measured by wearable actigraphy.

Actigraphy-measured deep sleep

The mean deep sleep duration was 88.78 ± 45.89 minutes among non-smokers compared with 81.10 ± 60.79 minutes among smokers (Figure 2). However, this difference did not reach statistical significance (t (95) =1.875, p=0.061). The mean difference between groups was 7.68 minutes, with a 95% CI ranging from −0.36 to 15.71 minutes (Table 1).

**Figure 3:**
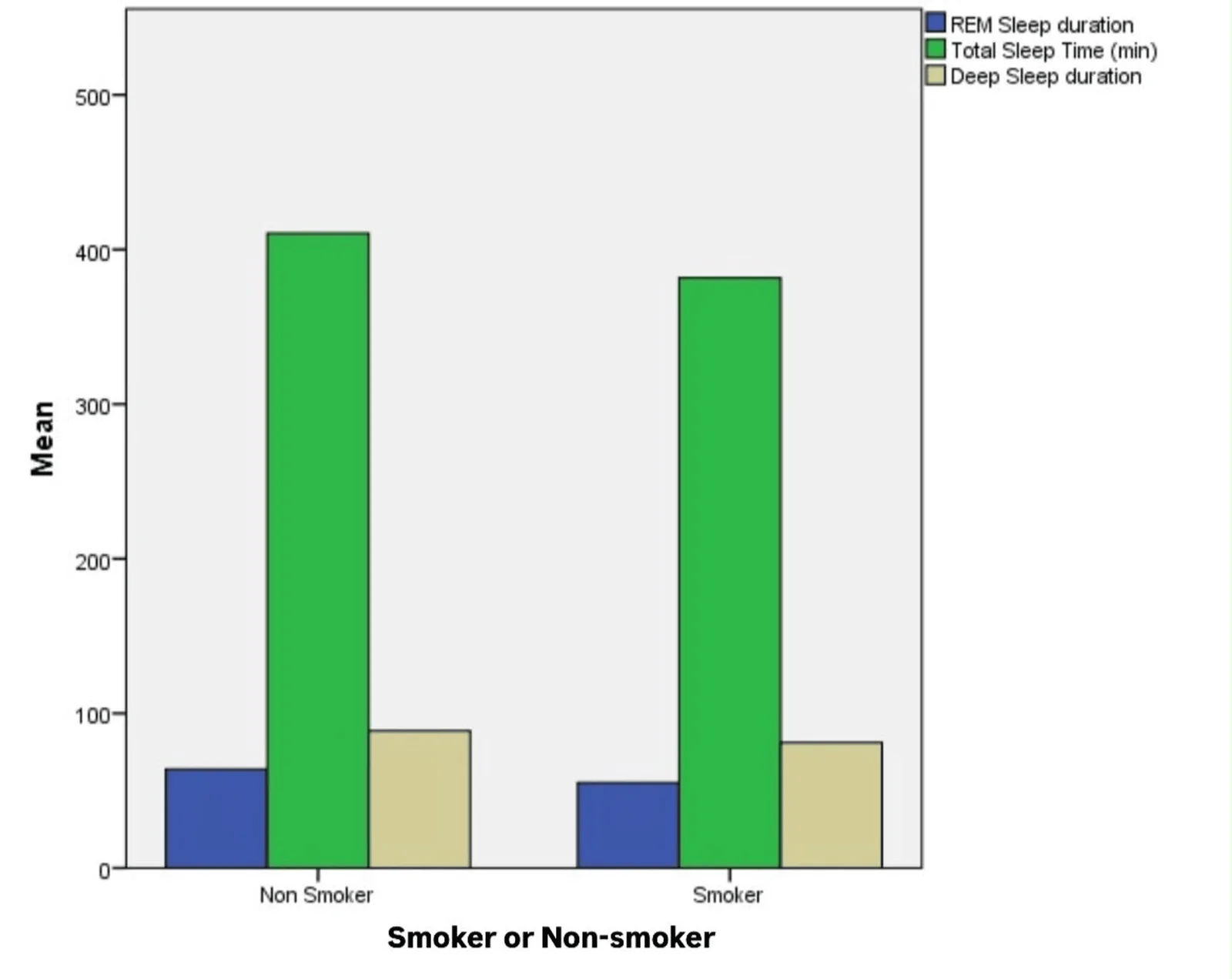
Comparison of mean REM sleep, deep sleep, total sleep time duration between smokers and non-smokers REM: rapid eye movement

Although smokers demonstrated a trend toward reduced deep sleep duration, the observed difference was not statistically significant (Figure 3).

Actigraphy measured nocturnal awakenings (≥3 minutes)

Nocturnal awakenings lasting ≥3 minutes were recorded using wearable actigraphy devices. However, awakening events demonstrated substantial night-to-night variability and occurred inconsistently across participants. Several participants demonstrated minimal or no awakening episodes, whereas others showed irregular brief awakenings.

Because awakening events were infrequent and did not demonstrate a normal distribution, reliable weekly mean values could not be calculated. Therefore, formal statistical comparison between smokers and non-smokers was not performed for this parameter.

Descriptive evaluation of the actigraphy recordings suggested generally low frequencies of nocturnal awakenings in both groups without a clearly observable between-group difference.

Summary of actigraphy findings

Overall, smokers demonstrated significantly shorter total sleep duration and significantly reduced REM-SD compared with non-smokers. Smokers also demonstrated a non-significant trend toward reduced deep sleep duration. Nocturnal awakening data could not be statistically analyzed because of variability and inconsistent event distribution.

Collectively, these findings suggest that smoking was associated with measurable alterations in objectively recorded sleep parameters, particularly total sleep duration and REM sleep duration.

Self-reported sleep habits 

Participant Characteristics

A total of 107 university students completed the survey and were included in the analysis. The sample consisted of 73 males (68.2%) and 34 females (31.8%), with a mean age of 21.65 ± 1.83 years (range: 18-28 years). Participants were recruited from more than 20 universities. The largest proportion was enrolled at the University of Georgia (n = 69, 64.5%), followed by Tbilisi State Medical University (n = 6, 5.6%), Caucasus International University (n = 3, 2.8%), Georgian National University SEU (n = 3, 2.8%), and Georgian Technical University (n = 3, 2.8%). The remaining participants were distributed across multiple institutions, each represented by one or two students (Table 2).

**Table 2:** Sociodemographic data of the survey participants

Characteristic	n (%)
Age, mean ± SD (years)	21.65 ± 1.83
Age range (years)	18–28
Sex	
Male	73 (68.2%)
Female	34 (31.8%)
University	
University of Georgia	69 (64.5%)
Tbilisi State Medical University	6 (5.6%)
Caucasus International University	3 (2.8%)
Georgian National University SEU	3 (2.8%)
Georgian Technical University	3 (2.8%)
Other universities	23 (21.5%)

Smoking Behavior

Prevalence and type of nicotine use: Current nicotine use was reported by 96 participants (89.7%), while 11 participants (10.3%) identified as non-smokers. Among the total sample, 48 students (44.9%) reported exclusive cigarette smoking, 28 students (26.2%) reported exclusive e-cigarette use, and 31 students (29.0%) reported dual use of both cigarettes and e-cigarettes.

**Figure 4:**
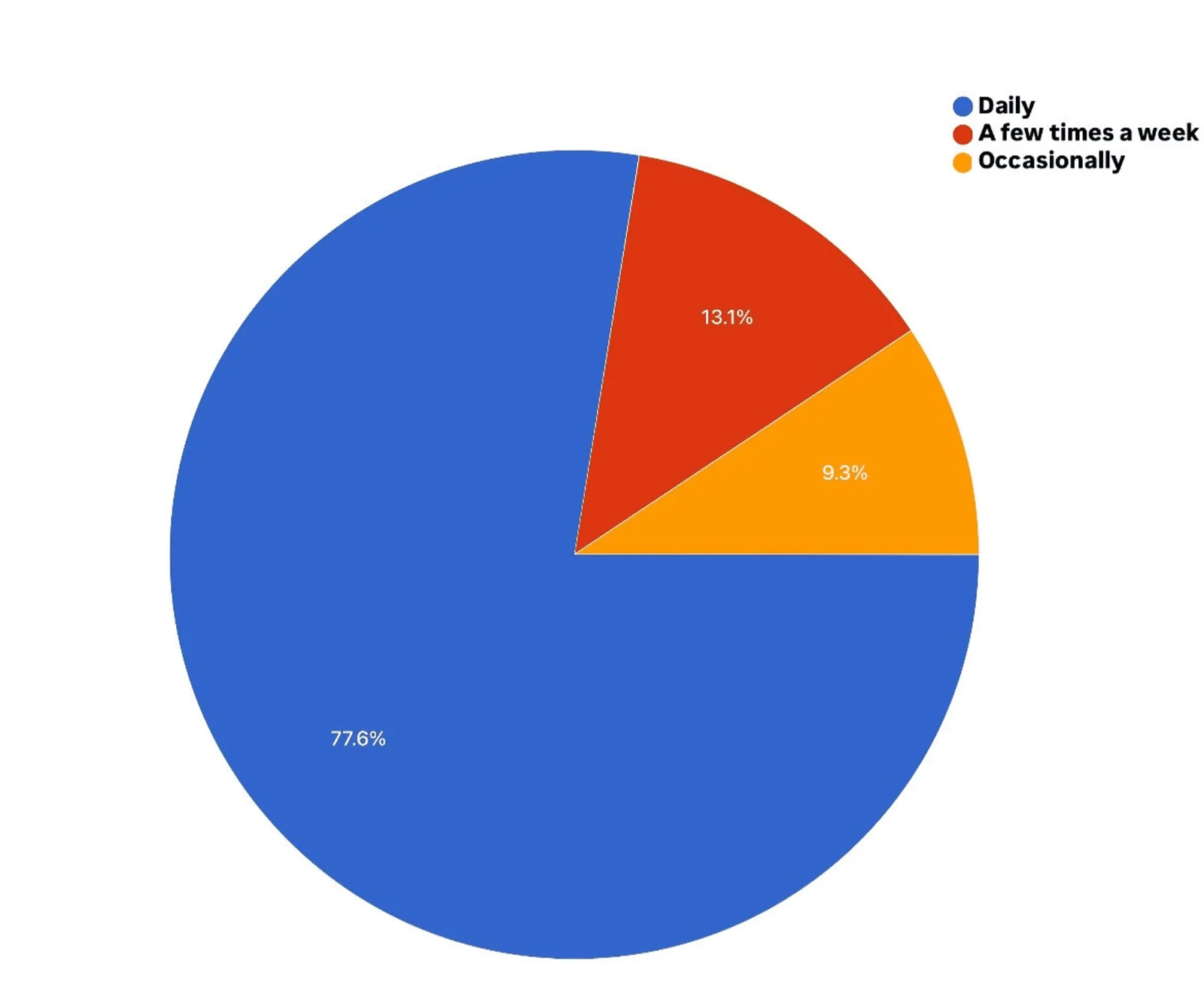
Frequency of smoking among the participants

Frequency and duration of use: Daily nicotine use was reported by 83 participants (77.6%), whereas 14 participants (13.1%) reported smoking a few times per week and 10 participants (9.3%) reported occasional use. Most smokers reported a smoking duration of six months or longer (n = 75, 70.1%), while 21 participants (19.6%) reported smoking for less than six months. Daily cigarette smokers reported consumption ranging from one to 30 cigarettes per day, with most reporting between five and 20 cigarettes daily (Figure 4).

Use of additional tobacco products: A minority of participants reported the use of other tobacco or nicotine products, including hookah, cigars, and nicotine pouches. These products were used infrequently and primarily in combination with primary nicotine sources.

Nicotine concentration among e-cigarette users: Among participants who used e-cigarettes (including exclusive and dual users; n = 59), reported nicotine concentrations varied. Thirteen participants (22.0%) reported using low concentrations (0-6 mg/mL), 17 participants (28.8%) reported moderate concentrations (12-18 mg/mL), 18 participants (30.5%) reported higher concentrations (20-30 mg/mL), and six participants (10.2%) reported very high concentrations (50 mg/mL). Five participants (8.5%) were uncertain of the nicotine strength used.

Smoking and Sleep Behavior

Smoking near bedtime: Smoking close to bedtime was commonly reported. Seventy-three participants (68.2%) reported smoking within two hours of bedtime, and 36 participants (33.6%) reported smoking within 30 minutes of going to sleep.

**Figure 5:**
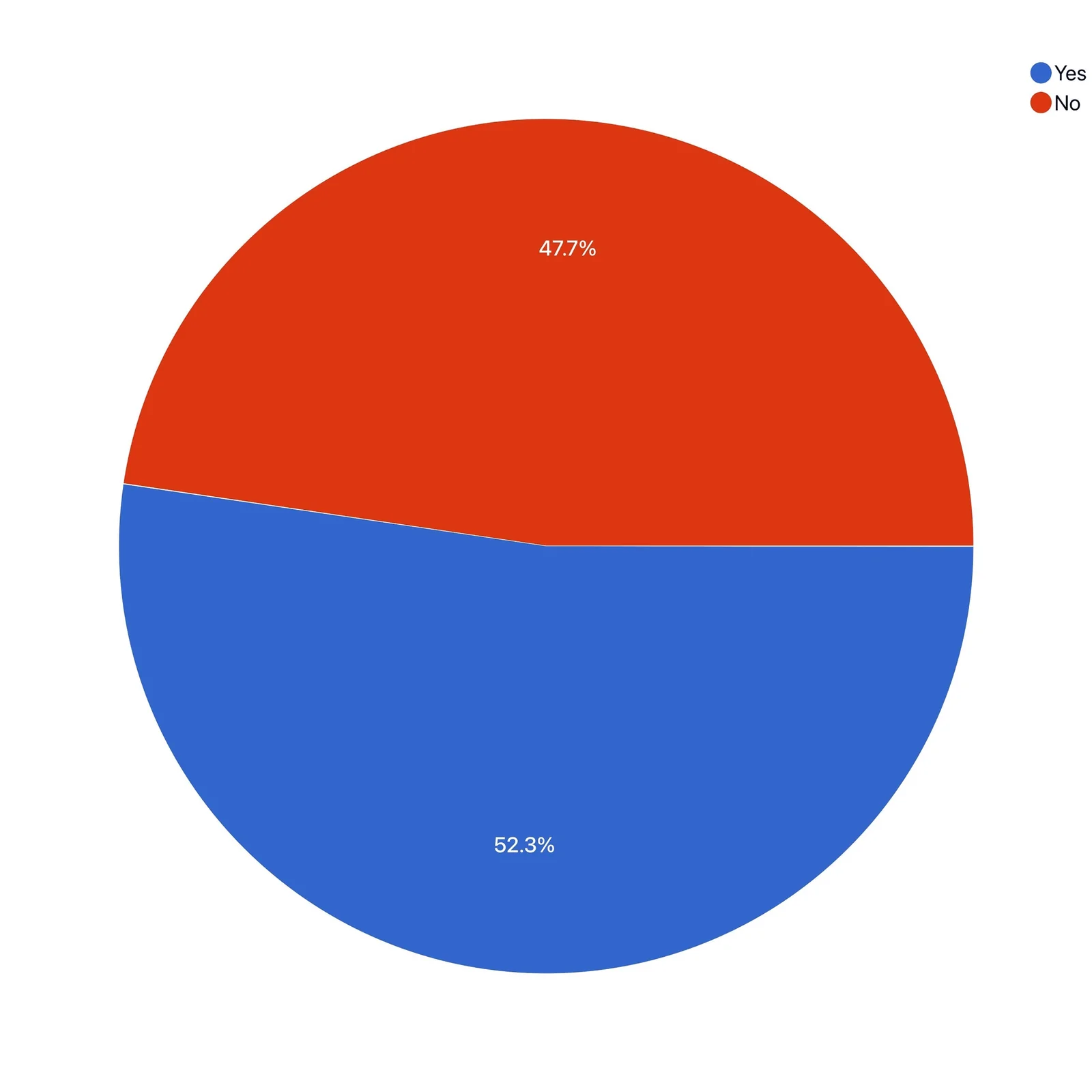
Self-reported effects of smoking on restful sleep

**Figure 6:**
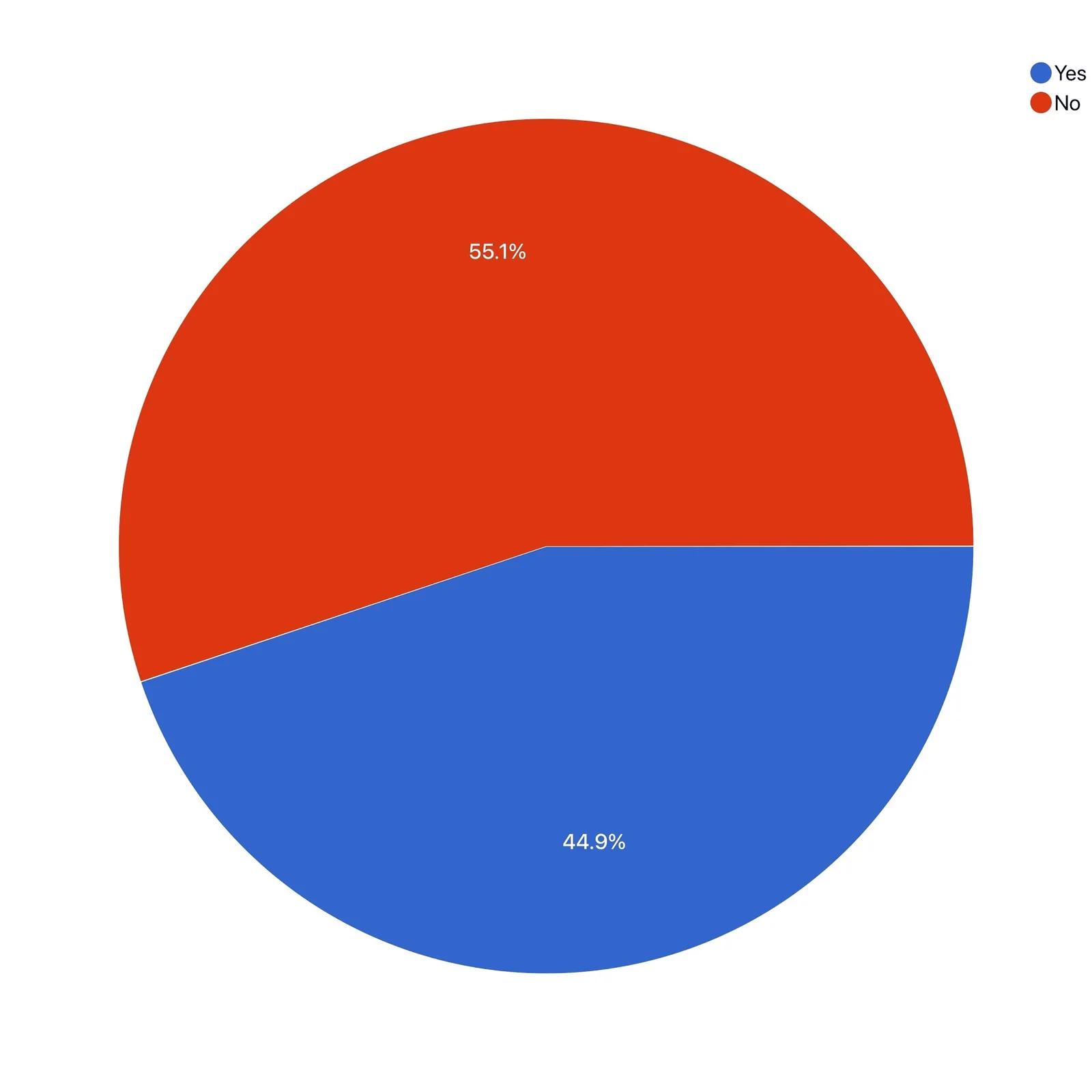
Self-reported morning restfulness among participants

Participants reported several sleep-related effects associated with smoking near bedtime, including delayed sleep onset (n = 71, 66.4%), restless or light sleep (n = 65, 60.7%) (Figure 5), nighttime awakenings (n = 51, 47.7%), and reduced morning restfulness (n = 74, 69.2%) (Figure 6). Nighttime nicotine cravings were reported by 21 participants (19.6%), with affected individuals reporting one to three awakenings per night.

Reasons for Smoking Before Sleep

**Figure 7:**
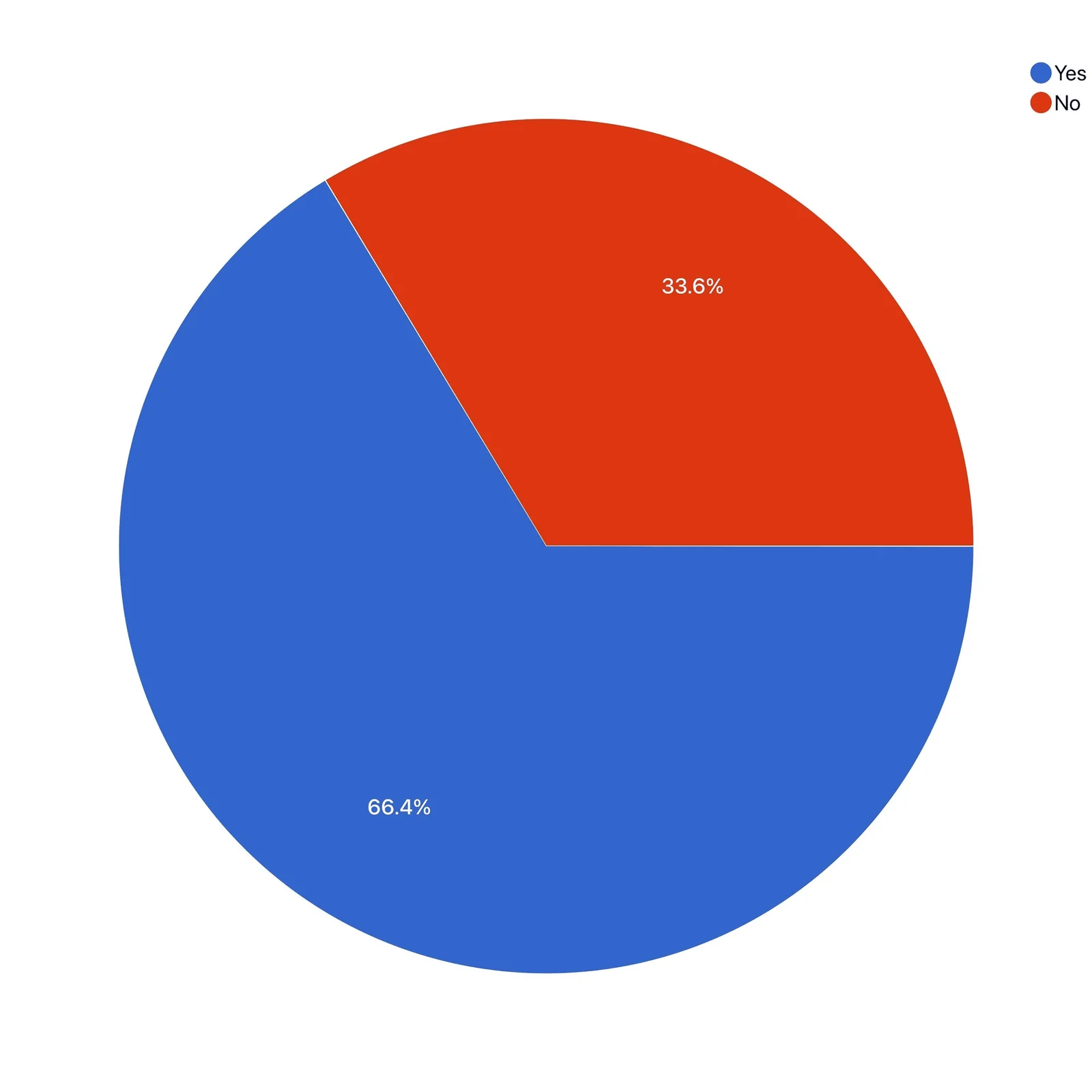
Reasons for smoking before bedtime

The most frequently reported reasons for smoking before bedtime included stress relief (n = 65, 60.7%), habitual behavior (n = 60, 56.1%), seeking relaxation (n = 52, 48.6%), and anxiety or difficulty winding down (n = 45, 42.1%). Multiple responses were permitted (Figure 7).

Sleep Characteristics and Fatigue

Reported sleep duration most commonly ranged between five and eight hours (n = 88, 82.2%). Poor or inconsistent sleep quality was reported by 69 participants (64.5%), and 76 participants (71.0%) reported experiencing morning fatigue. Difficulty maintaining sleep was reported by 58 participants (54.2%), while 49 participants (45.8%) reported early morning awakenings. Feeling unrefreshed despite adequate time in bed was reported by 74 participants (69.2%).

Vivid dreams or nightmares were reported by 40 participants (37.4%), often occurring during periods of increased stress or nicotine use. Irregular sleep schedules were reported by 81 participants (75.7%).

Stress, Lifestyle Factors, and Sleep

Academic stress affecting sleep was reported by 79 participants (73.8%), with 72 participants (67.3%) reporting increased smoking during periods of heightened stress. Screen use before bedtime was reported by 87 participants (81.3%), and low levels of physical activity were reported by 66 participants (61.7%). A smaller proportion of participants (n = 15, 14.0%) reported night shifts or irregular work or study schedules.

Impact of Smoking on Sleep Quality

Participants reported multiple sleep disturbances attributed to smoking, particularly when nicotine was used later in the day. These included difficulty falling asleep (n = 71, 66.4%), shortened sleep duration (n = 57, 53.3%), lighter or non-restorative sleep (n = 65, 60.7%), and frequent nocturnal awakenings (n = 51, 47.7%). Symptoms consistent with nicotine withdrawal affecting sleep, such as restlessness and difficulty maintaining sleep, were reported by 44 daily smokers (41.1%).

Study Compliance and Consent

Most participants reported willingness to comply with objective sleep monitoring, with 97 participants (90.7%) indicating they were willing to wear an actigraph device throughout the study period. Medical conditions that could influence sleep (including anxiety disorders, chronic pain, or insomnia) were reported by 13 participants (12.1%). Willingness to abstain from nicotine for at least two hours before bedtime was reported by 67 participants (62.6%). Full informed consent for sleep data collection and analysis was provided by 103 participants (96.3%).

Summary of survey findings

This survey-based component of the study evaluated smoking behaviors and sleep-wake characteristics among 107 university students to contextualize assessment of sleep. Nicotine use was highly prevalent, with the majority of participants reporting current and frequent smoking, including cigarettes, e-cigarettes, and dual use. Daily nicotine consumption and smoking close to bedtime were common practices, indicating sustained exposure during periods critical for sleep-wake regulation.

Sleep disturbances were frequently reported and showed a consistent temporal association with smoking behavior, particularly evening and pre-bedtime nicotine use. Participants commonly described delayed sleep initiation, increased nocturnal awakenings, lighter and less restorative sleep, and reduced morning alertness. Despite reporting sleep durations within an expected range for young adults, many participants experienced persistent fatigue and poor perceived sleep quality, suggesting impaired sleep continuity rather than reduced sleep opportunity.

Lifestyle and academic factors further influenced sleep-wake patterns. High academic workload, irregular schedules, late-night screen exposure, and low physical activity levels were widespread and often accompanied by increased smoking frequency. Nicotine use was frequently reported as a coping strategy for stress and relaxation; however, many participants also reported worsening sleep during periods of increased intake or withdrawal, highlighting the bidirectional relationship between smoking and sleep disruption.

Overall, the survey findings demonstrate a clear association between smoking behavior and disruption of the sleep-wakefulness cycle in university students. These results support the need to quantify alterations in sleep timing, continuity, and circadian regulation associated with nicotine exposure. Integrating smoking cessation strategies with sleep health interventions may be essential for improving sleep-wake outcomes in student populations.

## Discussion

In the actigraphy cohort, this study provides strong evidence that smoking, including both conventional cigarette use and e-cigarette use, is associated with increased risk and effects on objectively measured sleep among university students. It is now more focused on the macrostructure of sleep, which includes the REM and NREM sleeping patterns. The findings indicate that there are disturbances to the quantitative and qualitative sleeping patterns. There is a consistent experience of smokers with a shorter total sleep duration compared to nonsmokers. In addition to that, they also have shorter REM sleep, which can further lead to sleep problems that are particularly important for cognitive processing, memory consolidation, and emotional regulation. There is a trend towards reduced deep sleep within smokers, which is an indicative marker for potential compromise of the most restorative phase of sleep. No significant difference in sleep fragmentation or nocturnal awakening patterns was observed between smokers and non-smokers in the actigraphy cohort. All of this collectively indicates that exposure to nicotine is linked to both quantitative and qualitative alterations in sleep architecture. This, furthermore, results in overall poor sleep quality and a disturbed restorative sleep process among the university students. 

In our study, we used actigraphy. It is a portable wristband device that is used to measure movements [15]. It is a reliable tool to assess sleeping patterns [16]. It helps in assessing REM sleep, deep sleep, and light sleep, which are the basic parameters that we used in this research study. So, our study population has wrist-worn devices that measure and record daily information about lifestyle factors, sleep-wake parameters, and heart rate. They are sensitive to measuring REM sleep, deep sleep, wakefulness, total sleep duration, and fragmented sleep. This helped us to collect data about smokers and nonsmokers among university students. 

To our knowledge, there are no previous studies that have evaluated smoking of conventional cigarettes and e-cigarettes in relation to sleep architecture using actigraphy. Therefore, we started by searching for related articles and studies that correlate with our study. We found that from previous articles, smokers have an increased risk of suffering from severe insomnia [17], which correlates with our finding of decreased sleep quantity in the smoker population. According to another study, nicotine acts as a stimulant, raising heart rate and delaying REM sleep, which can lead to fragmented sleep and reduced deep and restorative stages [18-20], and that relates to our study where we found that smokers have mild fragmented sleep with decreased REM sleep. This can also increase the risk of sleep apnea due to the respiratory effects of tobacco. On the other hand, e-cigarettes or vapes have a higher odds of sleep-related complaints [7]. Among US young adults, e-cigarette users are more prone to short sleep duration [21], which correlates with the smokers' population in our study having significantly shorter total sleep duration, including the e-cigarette users. Also, according to another study, exclusive e-cigarette users and dual users were all at increased risk of inadequate sleep [22]. Like other stimulants, nicotine, which is a core compound in both cigarettes and e-cigarettes, has a presence that increases REM latency, sleep start latency, and NREM 2 sleep during acute intoxication. According to reports, chronic smokers are twice as likely to experience sleep problems, the most prevalent of which are increased sleep latency and daytime drowsiness. Chronic smokers' use of nicotine was also associated with higher REM latency and shorter total sleep duration, and our study has shown a reduction in REM sleep duration as a result; this may affect the normal sleep macrostructure. The study has also shown that the effects of withdrawal on sleep often start six to 12 hours after quitting nicotine, peak in one to three days, and last for up to three weeks. The effects include a decrease in REM latency and sleep onset latency, as well as an increase in REM sleep and wakefulness following sleep onset [23, 24]. This also adds to our study and supports the cessation of smoking among university students, which might reflect a good outcome by increasing REM sleep and total sleep duration. And they restore the normal sleep macrostructure and architecture.

In the separate survey cohort of 107 university students, the findings also demonstrated an association between smoking behavior and reported sleep characteristics, providing complementary evidence to the objectively measured actigraphy findings. 

These results occurred due to nicotine's action on acetylcholine receptors in the central nervous system (CNS) and autonomic ganglia, which stimulates sympathetic nervous system activity that leads to the release of neurotransmitters such as norepinephrine, dopamine, and acetylcholine, which obviously promotes wakefulness. It simultaneously decreases GABAergic inhibitory tone, reducing the transition into the stable sleep stages. Therefore, sleep onset latency is increased due to critical arousal and catecholamine activation. Total sleep time is decreased due to frequent arousals and awakenings. REM duration is decreased due to the inhibition of cholinergic neurons involved in REM generation. The nicotinic acetylcholine receptors (nAChRs) located in the CNS and autonomic ganglia are activated, which enhances arousal and attention, opposing the inhibitory neurophysiology mechanisms required for REM sleep. Sympathetic stimulation results in suppression of REM generation and fragmented sleep. Things to be noted are that chronic smokers have upregulation and desensitization of nAChRs due to the adaptation of the brain to continuous stimuli, which can increase wakefulness and reduce REM sleep. Now, when abstinence takes place, withdrawal symptoms such as anxiety, irritability, and restlessness can lead to fragmented sleep too and REM disruption. Smoking cessation might lead to REM rebound due to the decrease in nicotinic receptor activity, reducing sympathetic tone and allowing REM sleep to return to normal duration with shortened REM latency. This supports the fact that smoker students in university must be advised to stop smoking, thereby increasing their total sleep duration, increasing REM sleep duration, and shortening REM latency, which results in better cognitive functions and better memory capacity. 

In addition, the survey findings that included 107 university students showed a clear association between smoking behavior and sleep distribution. These findings may indicate that nicotine exposure can contribute to disturbances in normal circadian regulation and sleep-wake patterns, potentially affecting the timing, duration, and continuity of sleep.

This study allows us to raise awareness and demonstrate the severity of the dangers of smoking for both smokers and non-smokers. One of the reasons for the negative impact on sleep quality is the potential for poor concentration during study sessions and general discomfort, as smokers do not get the rest they need. Our study reinforces the importance of quitting smoking and its impact on social and psychological well-being, emphasizing the fundamental human right to restful and complete sleep. The average total sleep duration and differences between smokers and non-smokers are presented in the Results section, including a detailed breakdown of the 47 smokers and 50 non-smokers included in the study. Table 1 further illustrates sleep quality. It is worse for smokers, who are a major cause of discomfort in daily routines.

In the limitations of the study, we encountered several problems with some participants regarding the information we received about sleep and deep sleep. Several factors can negatively impact the study, most notably excessive, moderate, and light smoking among some participants. We excluded individuals who were not providing accurate information. However, the most significant factors that can impair sleep quality and lead to inaccurate information are stress, anxiety, noise, inadequate sleeping hours, caffeine, and alcohol. These are all key factors that can significantly impair sleep quality. We reinforced the study to prevent those factors with a daily reminder to the participants and key points to follow during the study to minimize bias. Alcoholic participants were not included. As this was a cross-sectional study, the observed associations between smoking and sleep characteristics cannot establish a causal relationship. Therefore, it cannot be determined whether smoking directly contributed to alterations in sleep or whether other unmeasured or residual factors may have influenced the observed associations. In addition, the study population consisted of university students aged 18-25 years, which may limit the generalizability of the findings to other age groups and populations. The findings should therefore be interpreted within the context of a young university-student population. Despite these limitations, the study has several strengths. The use of wrist-worn actigraphy provided objective assessment of sleep-wake patterns and allowed comparison of sleep characteristics between smokers and non-smokers. In addition, the study evaluated both conventional cigarette and e-cigarette use and incorporated a separate survey cohort, providing complementary information on smoking behavior and sleep characteristics. The use of clearly defined inclusion and exclusion criteria and monitoring over seven consecutive days further strengthened the reliability of the actigraphy data. 

Future studies must show clear and strict criteria for participants and a controlled environment, as environmental factors can distort the accuracy of information, as explained in the previous section on study limitations. We have already outlined the most important factors. Furthermore, the study should avoid including individuals living under constant stress and pressure. It should also differentiate between heavy smokers, moderate smokers, and light smokers. Additionally, participants must not use multiple tobacco products simultaneously, which may provide information based on personal bias. The study should also avoid including individuals with alcohol addiction or those who regularly consume energy drinks. It is important to monitor individuals who have quit smoking, as differences in sleep quality, duration, continuity of sleep, and sudden awakenings can be observed. Another major reason for inaccurate sleep data is that some individuals may experience persistent anxiety related to school or university assignments, or they may have other health problems. In the workplace, it is important to study and continuously record the environment of the person involved. All these factors can stimulate excessive thinking and result in fewer hours of sleep than usual. Remembering times and all details related to the research is essential, as providing intricate details is important.

The findings of this study have important implications for university students and public health. The observed association between smoking and altered sleep characteristics highlights sleep disturbance as an additional potential consequence of smoking among young adults. These findings may support the inclusion of sleep health in smoking-cessation education and counseling, particularly among university students. Healthcare professionals and university health services may consider addressing sleep patterns when discussing the broader effects of smoking and the potential benefits of smoking cessation. Furthermore, the findings may contribute to increased awareness among students regarding the potential relationship between tobacco use and sleep health. However, given the cross-sectional design, these implications should be interpreted in terms of association rather than causation.

## Conclusions

Finally, the present study offers objective proof that smoking is linked to sleep disturbances in university students. A history of cigarette smoking and use of e-cigarettes was associated with lower sleep quality, including decreased total sleep time and REM sleep, indicating that nicotine may adversely affect aspects of normal sleep architecture. The results are significant in that they suggest that smoking affects not only health risks but also the role of sleep in restorative and regulatory processes. In young adults, these disruptions could lead to suboptimal cognitive functioning, concentration, and emotional health, and result in a loss of capacity to function normally in daily life and to achieve academic success. Overall, the findings confirm that there is a case for promoting smoking cessation as a significant element of promoting sleep health in university students. Nicotine cessation and sleep hygiene education delivered as public health interventions can be particularly beneficial in decreasing sleep disruption and enhancing immediate functioning and future health outcomes.

## Appendices

Appendix A

Study Questionnaire

Effects of Smoking on the Sleep-Wakefulness Cycle: Actigraphic Research in University Students

This questionnaire was administered to university students to assess demographic characteristics, smoking behavior, sleep habits, and perceived effects of smoking on sleep.

Participant Information

Gender

• Male

• Female

Age

• Free-text response

University of Study

• University of Georgia

• Ilia State University

• TSU

• TSMU

• CIU

• NVU

• Other

Section 1: Smoking Behavior

1. Do you currently smoke cigarettes or e-cigarettes (vape)?

• Yes

• No

2. Which do you consume?

• Cigarettes

• E-cigarettes (vape)

• Both

2.1 If you use vape, what is the strength of nicotine in it?

• 0 mg/ml

• 3 mg/ml

• 6 mg/ml

• 12 mg/ml

• 18 mg/ml

• 20 mg/ml

• Other

3. How often do you smoke?

• Daily

• A few times a week

• Occasionally

4. If daily, how many cigarettes do you smoke per day?

• Free-text response

5. Have you been smoking for at least the past six months?

• Yes

• No

6. Do you use any other tobacco products (e.g., cigars, hookah, smokeless tobacco)?

• Yes

• No

7. If yes, which type of product?

• Cigars

• Hookah

• Smokeless Tobacco (SNUS)

• Other

Section 2: Smoking & Sleep Behavior

8. Do you smoke within two hours before going to bed?

• Yes

• No

9. Have you noticed a difference in your sleep quality when you smoke closer to bedtime?

• Yes

• No

10. Do you wake up during the night with cravings for nicotine?

• Yes

• No

11. If yes, how many times?

• Free-text response

12. Do you use any recreational drugs, including marijuana?

• Yes

• No

13. Do you consume nicotine in any form other than cigarettes or e-cigarettes?

• Yes

• No

14. Have you tried reducing or quitting smoking in the past? If yes, did it affect your sleep?

• Free-text response

Section 3: Exclusion of Other Substances

15. Have you used marijuana (cannabis) in the past three months?

• Yes

• No

16. Do you consume caffeine (e.g., coffee, energy drinks) within 4 hours before bedtime?

• Yes

• No

17. Do you take any prescription or OTC medications that affect sleep (e.g., sleeping pills, stimulants, antidepressants)?

• Yes

• No

18. If yes, please mention the type of prescription below:

• Free-text response

19. Do you regularly consume alcohol?

• Yes

• No

20. If yes, how often?

• Daily

• Weekly

• Occasionally

21. In the past month, have you consumed alcohol within two hours of bedtime at least twice a week?

• Yes

• No

Section 4: General Sleep Habits

22. Do you have a regular sleep schedule on most days of the week?

• Yes

• No

23. On average, how many hours of sleep do you get per night?

• Less than 4 hours

• 4-6 hours

• 6-8 hours

• More than 8 hours

24. How long does it usually take you to fall asleep?

• Less than 15 minutes

• 15-30 minutes

• 30-60 minutes

• More than 60 minutes

25. How often do you wake up during the night?

• Never

• 1-2 times

• 3-4 times

• More than 4 times

26. Do you experience difficulty falling asleep after waking up during the night?

• Yes

• No

27. Do you wake up feeling well-rested in the morning?

• Yes

• No

28. Do you feel excessively sleepy during the day?

• Yes

• No

29. Do you have any diagnosed sleep disorders (e.g., insomnia, sleep apnea)?

• Yes

• No

30. If yes, which one?

• Insomnia

• Sleep Apnea

• Restless Legs Syndrome

• Other

31. Do you frequently take naps during the day?

• Yes

• No

32. If yes, how many?

• Free-text response

33. Do you work night shifts or have irregular work/study hours?

• Yes

• No

Section 5: Impact of Smoking on Sleep

34. Do you experience vivid dreams or nightmares when you reduce or stop smoking?

• Yes

• No

35. Have you noticed increased restlessness or difficulty staying asleep when trying to quit smoking?

• Yes

• No

36. If yes, what is the duration of your sleep? (in hours)

• Free-text response

37. Have you ever noticed differences in your sleep quality on nights when you smoke more than usual?

• Yes

• No

38. Do you believe smoking affects your ability to get deep, restful sleep?

• Yes

• No

39. Have you ever used smoking as a way to help you relax before bed?

• Yes

• No

40. Do you think nicotine withdrawal symptoms (e.g., irritability or restlessness) affect your sleep?

• Yes

• No

41. If you smoke more during stressful periods, do you also notice changes in your sleep quality?

• Yes

• No

42. If yes, in your own words, explain what type of changes you notice in your sleep quality:

• Free-text response

Section 6: Additional Information & Consent

43. Have you participated in any sleep studies before?

• Yes

• No

44. Would you be able to wear an actigraph device continuously for the study period?

• Yes

• No

45. Do you have any medical conditions that could affect your sleep patterns (e.g., chronic pain, anxiety disorders)?

• Yes

• No

46. If yes, please mention the type of disorder/medical condition below:

• Free-text response

47. Are you willing to avoid nicotine use at least two hours before bedtime during the study?

• Yes

• No

48. Do you give consent for your sleep data to be collected and analyzed?

• Yes

• No
